# Curing Regime-Modulating Insulation Performance of Anhydride-Cured Epoxy Resin: A Review

**DOI:** 10.3390/molecules28020547

**Published:** 2023-01-05

**Authors:** Jin Li, Hein Htet Aung, Boxue Du

**Affiliations:** Key Laboratory of Smart Grid of Education Ministry, School of Electrical and Information Engineering, Tianjin University, Tianjin 300072, China

**Keywords:** electrical and electronic equipment, insulation performance, bisphenol-A epoxy resin, anhydride, curing regime

## Abstract

Anhydride-cured bisphenol-A epoxy resin is widely used in the support, insulation and sealing key components of electrical and electronic equipment due to their excellent comprehensive performance. However, overheating and breakdown faults of epoxy resin-based insulation occur frequently under conditions of large current carrying and multiple voltage waveforms, which seriously threaten the safe and stable operation of the system. The curing regime, including mixture ratio and combination of curing time and temperature, is an important factor to determine the microstructure of epoxy resin, and also directly affects its macro performances. In this paper, the evolution of curing kinetic models of anhydride-cured epoxy resin was introduced to determine the primary curing regime. The influences of curing regime on the insulation performance were reviewed considering various mixture ratios and combinations of curing time and temperature. The curing regime-dependent microstructure was discussed and attributed to the mechanisms of insulation performance.

## 1. Introduction

Epoxy resin has been applied in the field of electrical insulation since the early 1950s. After more than 70 years of development, it has become a widely used insulating material in electrical equipment due to its excellent heat resistance, mechanical and insulation properties [1,2,3,4,5]. In gas-insulated metal-enclosed lines (GIL) and gas-insulated metal-enclosed switches (GIS), basin insulators or tri-post insulators made of epoxy resin as the main raw material through the casting process play a major role in supporting and insulating, and are key components of GIL/GIS foundation units [6,7]. The fiber-impregnated epoxy resin-cured insulation pipe, due to its good tensile and breaking strength, constitutes the main functional structure of the insulation rod [8]. In the high-voltage converter bushing, the epoxy-impregnated paper (RIP) insulation core formed by wrapping the conductor with corrugated paper and curing it with epoxy resin is the core of the entire bushing, which bears the main heat dissipation, mechanical load and ground insulation of the equipment [9]. High frequency transformers adopt epoxy resin composite material to realize overall insulation of equipment, which can reduce insulation distance, reduce equipment volume and improve power density [10]. It can be seen that epoxy resin has been widely used in various electric power applications and types of electronic equipment as an electrical material that undertakes the main insulation function.

With the complication of high-voltage equipment application scenarios, high-temperature, high-field, multi-voltage waveform and other operating conditions affect the thermal and insulation properties of epoxy resin. Thermodynamic calculation under relevant allowable load shows that the maximum temperature of epoxy-impregnated paper insulation core of transformer bushing can reach 107 °C, and there is a temperature gradient of 12.5 °C/cm [11]. The increase of temperature will lead to the deterioration of the insulation performance. The conductivity of epoxy resin increases exponentially with the increase of temperature. The conductivity at 100 °C can increase by nearly three orders of magnitude compared with that at 25 °C; the dielectric loss increases with the increase of dielectric constant at high temperature; the short-time breakdown field strength can be reduced by 61.39% from 30 °C to 150 °C, and the breakdown time under long-term constant voltage can also be significantly reduced at high temperatures [12,13,14]. The voltage waveform of a high frequency transformer with a large capacity is a square-like wave containing complex high-frequency harmonics. The dielectric loss of epoxy resin increases when harmonics exist, and the harmonic stress will increase the risk of insulation breakdown. Under the effect of long-term high voltage, harmonics and high temperature, the insulation material may have complex deterioration and aging characteristics [15,16,17]. The complex and changeable operating environment puts forward higher performance requirements for electrical epoxy resin.

The cured product of epoxy resin is formed by the long-term curing of epoxy resin matrix and curing agent under the conditions of adding relevant additives and specific ambient temperature and is applied to relevant electrical equipment. After the curing process, the epoxy resin system is cross-linked by liquid mixture to form a three-dimensional network structure. The microstructure of epoxy resin determines its macro comprehensive performance and affects its operating state in the working environment. The curing degree, crosslinking density, free volume and other indicators can reflect the structural characteristics of epoxy resin. Relevant research shows that with the increase of curing degree, the network size of epoxy resin gradually increases, the mobility of free ions decreases, and the influence of dipole relaxation increases, leading to changes in dielectric properties [18]. The decrease of crosslinking density indicates that there are more side chains and branches in the epoxy crosslinking network, which leads to the increase of dielectric constant and the decrease of thermal stability [19]. The free volume affects the dielectric properties and breakdown characteristics of epoxy resin. The increase of free volume provides more space for the activity of polar groups, making it easier for the dipole to follow the change of external electric field, which usually leads to the increase of dielectric constant [20]. At the same time, the increase of the free volume will lead to the increase of the average free path of the electron under the action of the electric field and the increase of the accumulated energy, which will more easily damage the molecular chain and lead to breakdown. The microstructure of epoxy resin is closely related to the system components and curing system. In order to improve the comprehensive performance of epoxy resin, adding filler to the original system has become a key research direction. Different fillers are used to improve the thermal, mechanical, insulation and other properties of epoxy resin to meet the needs of different situations [21,22,23]. However, the performance of epoxy resin is not only related to the properties of its own substances, but also to the process conditions during curing [24]. The cured products of epoxy resin formed under a reasonable and scientific curing system can fully reflect the good performance of its bulk components, which is also an effective technical means to improve the performance of epoxy resin. However, the research content of the epoxy curing system is less than filler modification, and less attention is paid to the influence of the curing system on insulation performance.

This paper will systematically summarize the influence of the curing system on the insulation performance of epoxy resin, which is of great value and significance for understanding the crosslinking principle and the corresponding relationship between structure and insulation performance of epoxy resin, optimizing the curing process of epoxy resin, and improving the performance of epoxy resin.

## 2. Reaction Mechanism of Anhydride-Cured Epoxy Resin

### 2.1. Bisphenol-A Epoxy Resin Matrix

**Figure 1:**
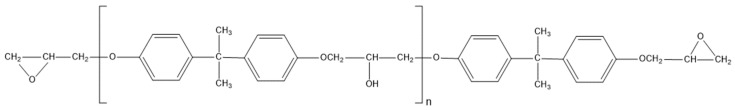
Chemical structure of bisphenol-A epoxy resin.

Epoxy resin is the general name of a class of compounds that contain two or more epoxy groups in the molecule and can form a three-dimensional cross-linking network structure with the participation of appropriate chemical reagents [25,26]. The epoxy group has high reactivity, which is attributed to the strong deformation ability of the ternary ring and the shift of the charge in the epoxy ring. The charge in the epoxy group is significantly biased towards the oxygen atom, that is, the electron cloud density around the oxygen atom is significantly greater than the other two carbon atoms, which results in the ring opening of the epoxy group caused by the attack of the nucleophilic reagent on the carbon atom and the attack of the electrophilic reagent on the oxygen atom [27]. There are many kinds of epoxy resin, and the classification method is also complex. According to the different chemical structures, it can be generally divided into glycidyl ethers, glycidyl esters, glycidyl amines, alicyclic groups, epoxides, and new epoxy resins [28]. Bisphenol-A based epoxy resin (DGEBA) has become the most widely used epoxy resin due to its convenient source and low processing cost [29,30,31]. It has low curing shrinkage, strong product stability, high bonding strength, good corrosion resistance and electrical insulation performance. Its chemical structure is shown in Figure 1, where n represents the degree of polymerization of the epoxy resin matrix, usually between 0~1. Bisphenol-A epoxy resin is a multi-molecular weight mixture composed of parts with different polymerization degrees. The properties of the epoxy resin come from the functional groups that make up each part of the epoxy resin: the epoxy groups at both ends of the molecule give high reactivity; The skeleton composed of benzene ring has strong rigidity, while a large number of methylene distributed on the main chain gives flexibility; Hydroxyl and ether bonds provide good wettability and adhesiveness, which makes them very suitable for wettability or solid sealing processes.

### 2.2. Anhydride Curing Agent

At room temperature, epoxy resin is usually a viscous liquid or brittle solid and only by adding the appropriate chemical agents for cross-linking reaction can insoluble thermosetting resin be formed, which has practical application value [25]. This kind of chemical agent that can react with epoxy resin is called the curing agent, and this kind of chemical reaction is called the curing reaction. According to different curing reaction principles, curing agents can be roughly divided into visible and latent types. The apparent curing agent can directly react with the epoxy resin at the room temperature, while the latent curing agent can be mixed with the epoxy resin at a certain temperature to maintain long-term storage stability. Once the external heat, light, humidity and other conditions change, the curing reaction will be carried out. The apparent curing agent can be divided into two types: additive polymerization type and catalytic polymerization type. The additive polymerization type curing agent directly polymerizes with the epoxy resin matrix after opening the epoxy ring and participates in the formation of the cross-linking network itself. This kind of curing agent has the widest application range, including polyamine curing agent, acid anhydride curing agent, phenolic curing agent, etc. However, after opening the epoxy ring in the form of cations or anions, the catalytic polymerized curing agent can promote the crosslinking of hydroxyl groups and other groups of the epoxy matrix with the epoxy group. It does not participate in the formation of the network structure itself, but only plays a catalytic role.

Polyamine and anhydride curing agents account for more than 90% of all curing agents and are the two most widely used curing agents. The polyamine curing agent first relies on the active hydrogen of the primary amine to open the ring of the epoxy group to form a secondary amine, and then further reacts with the epoxy group to form a tertiary amine, finally forming a complex cross-linking network [32,33]. The mechanical properties of the product are relatively balanced, but the heat resistance is poor. The acid anhydride curing agent relies on the hydroxyl group in the epoxy to open the ring of the acid anhydride to form a carboxylic acid, which further reacts with the epoxy group. At the same time, the hydroxyl group can also open the ring of the epoxy group to form a cross-linking network [34]. The reaction temperature between anhydride curing agent and epoxy resin is high. Compared with amine curing agent, its shrinkage effect during curing is weak, and the heat resistance and insulation performance of the product are more excellent, so it is widely used in the field of electrical insulation [35].

Common anhydride curing agents can be divided into aromatic anhydride, alicyclic anhydride, aliphatic anhydride, etc. [36]. Aromatic anhydride includes trimellitic anhydride (TMA), pyromellitic dianhydride (PMDA), etc. The cured product has good heat resistance and excellent electrochemical performance, but it is solid at room temperature and has poor processing performance; The aliphatic anhydrides include polymaleic anhydride, polyazelaic anhydride, etc. It has good toughness and impact properties due to the long chain of fatty group in the molecular structure. Because there is no benzene ring in the molecular structure of alicyclic anhydride, its weather resistance is better than that of aromatic anhydride; because most varieties are liquid at room temperature and have better processability than aromatic anhydride, they have become the most widely used anhydride curing agent. Methyl hexahydrophthalic anhydride (MHHPA) is an alicyclic anhydride curing agent commonly used in the electrical and electronic devices. It has the advantages of low melting point, good compatibility, low viscosity of cured products, long service life, high heat resistance, excellent high-temperature insulation performance, etc. It is commonly used in the impregnation of electrical equipment coils, pouring of electrical components, the sealing of semiconductors, and other applications.

### 2.3. Accelerator and Matching Technology

In addition to the epoxy resin matrix and curing agent, additives are also indispensable for different application scenarios and performance requirements, such as accelerator, thinner, coupling agent, etc. Accelerators are often mixed with curing agents that can react at high temperatures to improve reaction activity, reduce curing temperature and shorten reaction time [37]. The reactivity of the anhydride curing agent and the epoxy resin matrix is very low at room temperature, and the reaction usually takes place above 200 °C [38]. Almost all anhydride curing agents need to be used together with accelerants. Promoters can be divided into nucleophilic promoters, electrophilic promoters and metal carboxylate promoters according to the catalytic principle. The nucleophilic accelerator is generally a Lewis base, which has a dual catalytic effect on the epoxy/anhydride curing system, namely, it can catalyze both epoxy crosslinking and anhydride ring opening. Its catalytic principle is generally to first form an alkoxy anion to react with anhydride, then generate a carboxyl anion to react with epoxy, and then generate a new alkoxy anion. The reaction alternates and finally forms a polyester cross-linked structure. The stronger the alkalinity of Lewis bases, the smaller the steric hindrance of substituents, and the greater the catalytic activity. The electrophilic accelerator is generally a Lewis acid and its complex. The catalytic principle of such organic acids, alcohols, or phenols is that they first go through the complex state and then form a curing cross-linked structure. Some Lewis acids are stable in combination with epoxy resin and curing agent at room temperature, and can accelerate the reaction at high temperature, play a catalytic role, and have the characteristics of latent accelerator. The metal ions in the metal carboxylates have empty orbits in the early reaction stage and can form complexes with epoxy groups to achieve catalytic polymerization; in the later stage, with the exothermic effect of the reaction, metal carboxylates dissociate into carboxylic acid anions to achieve catalytic polymerization. Two different catalytic mechanisms make the cured products have both ester groups and ether bonds. Manganese, zinc, calcium, lead and other metal carboxylates are often used as accelerators in production. The matching of accelerator and epoxy/anhydride system is of great significance for improving the product performance of epoxy resin.

**Figure 2:**
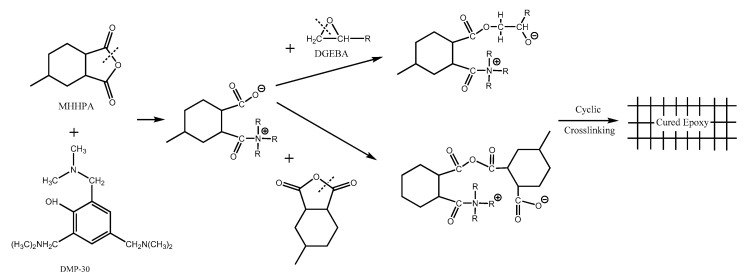
Reaction mechanism of DGEBA/MHHPA system catalyzed by DMP-30 [41].

2,4,6-tris-(dimethylaminomethyl) phenol (DMP-30) is an important accelerator often used in an epoxy resin/anhydride curing system [39]. In the epoxy/anhydride curing system, the tertiary amine in DMP-30 makes the anhydride group open the ring, forming anion and cation pairs, shown in Figure 2. The carboxyl anion is very easy to combine with the epoxy group to open the ring, causing further reaction, and finally forming a polyester cross-linking network; at the same time, the anion can also react with the anhydride group, and the ring-opening anhydride group continues to combine with the epoxy resin to continue to promote the construction of the cross-linking network. These two reactions will generate new anions, making the reaction continue [40,41]. The hydroxyl group can open the ring of anhydride or etherify the hydroxyl group directly with the epoxy group. The reactivity of tertiary amine with anhydride is stronger, and anions can be continuously generated later. Therefore, DMP-30 has significant catalytic effect, greatly reducing the reaction temperature of epoxy resin and anhydride curing agent. It is worth noting that in the presence of DMP-30, each epoxy group still reacts with an anhydride group, and the addition of accelerator does not change the optimal amount of curing agent. Because the content of accelerator in the system is very low, the main crosslinking structure is still composed of epoxy resin and curing agent. The function of the accelerator is only to improve the crosslinking speed of epoxy resin and curing agent.

## 3. Curing Kinetics

### 3.1. Characteristic Temperature

In the actual curing process, the epoxy resin is cured at a specific temperature, that is, the heating rate is 0 K/min. Therefore, the characteristic temperature of reaction at different heating rates can be obtained by linear fitting, which is the temperature extrapolation method. Results in Table 1 show that the characteristic temperature of the epoxy resin system showed a downward trend with the increase of accelerator content, which corresponded to the catalytic characteristics of the accelerator [41]. It is generally believed that the peak initial temperature corresponds to the temperature at which the system starts to react, and the peak temperature corresponds to the temperature at which the reaction is most intense. In the actual curing process flow, in order to prevent the initial reaction from being too fast and the exothermic reaction from being too violent, causing local temperature concentration and obvious thermal stress, the curing scheme first uses a lower temperature for curing for a period of time, and then increases the temperature to speed up the reaction to complete the curing [42]. Two-stage curing becomes a general curing scheme for epoxy resin. The pre-curing temperature is generally selected near the initial reaction temperature, and the post-curing temperature is generally selected as the peak temperature. According to the relevant fitting results, the pre-curing temperature of the system is determined to be 100 °C, and the post curing temperature is 140 °C.

### 3.2. Non-Model Fitting Curing Kinetics

The non-model fitting method is based on the assumption of equal conversion, that is, when the curing degree is the same, the reaction rate of the system is only related to temperature [43]. It avoids the premise that the model fitting method needs to assume the reaction mechanism function in advance and has a good fitting effect on complex reactions [44]. The essence of the non-model fitting method is to convert the integral or differential heat flow signal into the curing degree. The common non-model fitting methods include the Kissinger method, the Friedman method, the Flynn Wall Ozawa (FWO) method, etc. [45,46]. The non-model fitting method can predict the isothermal curing behavior of the system based on the non-isothermal test results, which is of great significance to guide the curing process, especially the curing time required for the epoxy resin to reach a specific curing degree at a specific temperature. Activation energy is a key parameter in the analysis of curing kinetics. It usually refers to the energy required for a molecule to change from an energy-stable ground state to an active state prone to chemical reactions. The apparent activation energy can measure the difficulty of chemical reaction. By comparing the activation energy obtained by different calculation methods, it can be seen from Table 2 that the reaction activation energy calculated by the Kissinger method and the FWO method is relatively close, which verifies the accuracy of the activation energy calculation and proves that the epoxy resin system follows the equal conversion criterion. In contrast, the Friedman method also follows the assumption of equal conversion rate, but the calculated activation energy is unstable, which is quite different from the values calculated by the other two methods. This is because the calculation method uses the real-time reaction rate dα/dt for fitting, and the reaction rate is greatly disturbed by environmental factors, making the data unstable.

### 3.3. Model-Fitting Curing Kinetics

The curing kinetics analysis can be divided into two categories according to whether it depends on the kinetic equation: the model fitting method and the non-model fitting method. The model fitting method assumes that the curing process conforms to a certain kinetic model. By fitting the kinetic parameters of the model, the relationship between curing rate, curing degree, time and temperature is established to reflect the law of the whole curing process. The fitting model can be divided into the mechanism model and the phenomenological model. The phenomenological model avoids the types and details of chemical reactions in the curing process, and directly reflects the thermodynamic law according to the exothermic form of the heat flow curve; the mechanism model derives the curing reaction model from the fundamental curing principle. Although the mechanism model can better predict and explain the curing process, due to the complexity of the curing reaction, it is very difficult to derive the mechanism model. At present, most studies still use phenomenological models. The commonly used phenomenological models of epoxy resin include the n-order reaction model, the Kamal model, the Sestak Berggren (SB (m, n)) model, etc. [47,48]. Bi et al. studied the applicability of the SB (m, n) model to the epoxy resin/anhydride/alumina system, and found that the fitting effect was good, and the kinetic parameters did not show a significant linear relationship with the increase of the curing agent content [49]. Ma et al. studied the effect of Kamal model fitting the epoxy/anhydride system, and the results proved that the reaction system is autocatalytic and is controlled by diffusion in the later stage of the reaction. The Kamal model cannot describe the reaction in this stage, and the theoretical value of the modified equation after introducing the diffusion factor is in good agreement with the experimental data [50]. Relevant research shows that the curing reaction of epoxy/anhydride system is autocatalytic, that is, the reacted part has a certain catalytic effect on the unreacted part. The reaction concentration is not the only factor determining the reaction rate, and the maximum reaction rate generally occurs in the middle period of the reaction [51,52].

The changes of the system morphology during the crosslinking process of epoxy resin can be roughly divided into different stages of liquid-gel-solid. Before the gel point, the epoxy resin system is generally in a liquid state with strong fluidity, and molecules everywhere in the system can effectively collide to initiate a cross-linking reaction, which is mainly controlled by the system concentration; after the gel point, the epoxy resin system is in a gel state, and its fluidity is weakened. The part with the higher concentration before the reaction is complete. The part with the lower concentration needs the remaining unreacted substances at the high concentration to diffuse to the low concentration before the reaction can occur. The crosslinking reaction depends on the flow ability of the unreacted part, and the reaction is mainly controlled by the system diffusion. The gel point is the key node for the change of the reaction process of the system, and the Kamal model focuses on the autocatalysis of the reaction process, that is, the reaction law of diffusion control; combined with the model fitting effect, it was found that the agreement was good after 60% reaction process. The fitting results show that the fitting effect is good before 60% of the reaction process, but there is a certain degree of deviation in the reaction process later, because the system in the post-reaction stage is controlled by diffusion, and the concentration is no longer the main factor affecting the reaction process. This is contrary to the fitting effect of the Kamal model. To sum up, the reaction of the epoxy resin of this system is controlled by concentration in the early stage, and then by diffusion. The dividing point is the gel point of the system, which is about 60% of the reaction process. The fitting effect of the n-order reaction model before gel point is better, while the fitting effect of the Kamal model after gel point is better yet, the model-fitting parameters of which are shown in Table 3. [36]

## 4. Crosslinking Structure Dependent Insulation Performance

There is a key problem in the study of curing system, that is, the quantitative determination of solidification degree. The degree of cure is usually defined as the ratio of the cross-linked epoxy group to the total epoxy group in epoxy resin, or the ratio of heat released by reaction to total heat released by reaction [53]. It is an important index to measure the reaction process of epoxy resin. It is of great significance to control the reaction process of epoxy resin, guide the production progress of epoxy resin, and regulate the cross-linking structure of epoxy resin to obtain the determined curing degree under the determined temperature and time conditions. There are two methods to determine the curing degree of epoxy resin: the thermokinetic method and infrared spectroscopy. The thermodynamic method obtains the reaction heat information of epoxy resin by DSC, and obtains the corresponding relationship between curing degree and time at a specific temperature by mathematical transformation between a kinetic equation or thermodynamic parameters [54]. This method has high accuracy and strong applicability, but the DSC test based on the liquid epoxy ingredients before curing belongs to prediction in advance, and the curing degree of cured samples cannot be determined. Although some researchers have developed online monitoring equipment based on sensor technology [55], it does not have universality. The infrared spectroscopy can calculate the curing degree of epoxy resin by quantifying the intensity of the epoxy group absorption peak obtained by the infrared spectroscopy. The curing degree of cured samples can be obtained, but the accuracy is low, and usually cannot meet the demand. How to quantify the curing degree of epoxy resin accurately and conveniently is still a key issue worthy of study.

The cross-linking structure of epoxy resin affects the insulation performance of cured products. Free volume, cross-linking density and other indicators are often used to reflect the changes of cross-linking structure. Free volume is considered to be closely related to dielectric properties and breakdown characteristics. Artbauer et al. found that the increase of free volume increases the free stroke of electrons in the polymer, which will lead to the decrease of insulation performance, especially the breakdown strength [56]. Fuad et al. studied the DC/AC breakdown strength of the epoxy resin/silicon nitride system and found that the free volume did not constitute the main factor affecting the breakdown strength, and the change of charge transport condition caused by the nano interface affected the breakdown strength of the system [57]. The increase of free volume usually leads to the increase of dielectric constant and dielectric loss, especially when the ambient temperature exceeds the glass transition temperature. Nascimento showed that the free volume of epoxy nanocomposite has a minor change compared with the pure epoxy system, which cannot well explain the change of dielectric behavior [58]. Guo et al. increased the cross-linking density of the epoxy resin network structure and effectively reduced the dielectric constant and dielectric loss by introducing the oriented structure of biphenyl mesocrystals [59]. The regulation of crosslinking structure is of great significance for improving the insulation performance of epoxy resin.

**Figure 3:**
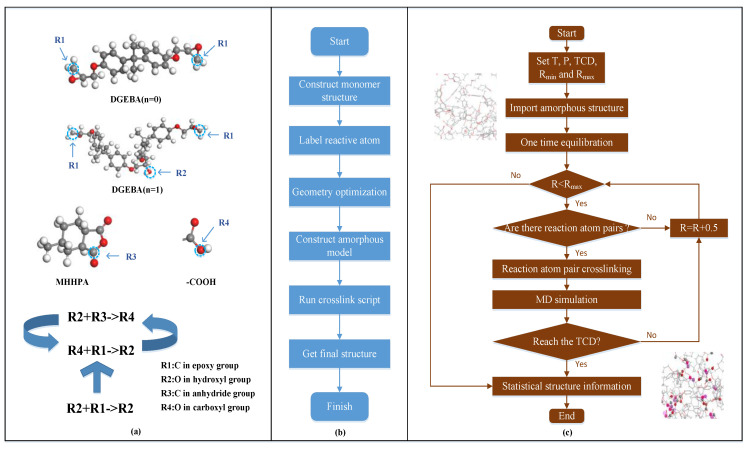
Simulation of epoxy resin crosslinking process (**a**) Atomic calibration names and reaction rules (**b**) Flow chart of structure construction (**c**) Flow chart of crosslinking script and partial structure before and after crosslinking [66].

Molecular dynamics simulation analyzes the statistical law of molecular motion through the high computing performance of the computer, obtains the molecular microstructure, reveals the relationship between molecular position conformation, motion state and macro performance indicators, and is widely used in the research field of epoxy resin [60,61]. How to simulate the reasonable and accurate crosslinking structure of epoxy resin by advanced technical means is a key problem in this field. At present, the crosslinking model of epoxy resin is mostly designed based on the “distance judgment criterion”, that is, searching for a pair of reactive atoms within the set reaction radius, conducting molecular dynamics equilibrium after crosslinking to obtain a stable conformation, and then continuously increasing the reaction radius until the relevant conditions are met [62,63]. Most of the existing epoxy resin crosslinking models are based on direct crosslinking between reaction atoms. For example, the crosslinking reaction between epoxy resin and anhydride curing agent is to directly generate a chemical bond between the calibrated carbon atom and oxygen atom [64]. However, the process of curing bisphenol A epoxy resin with anhydride involves multiple competitive reactions of esterification and etherification, so the direct interatomic reaction lacks certain rationality [65]. The design of more reasonable and scientific cross-linking rules to reveal the relationship between cross-linking structure and insulation performance is of great help to deepen the research on the curing system of epoxy resin. The crosslinking script of the epoxy resin system was developed by molecular simulation, shown in Figure 3, and its validity was verified by glass transition temperature. The effects of curing degree on the DC conductivity, dielectric constant, dielectric loss, and AC breakdown strength of epoxy resin were analyzed by using the simulated epoxy cross-linking structure characteristics, energy level orbit and electrostatic potential distribution, and the dependence of epoxy resin structure evolution on insulation performance was explored [66].

## 5. Curing Regime

The performance of epoxy resin depends on its cross-linking structure. The properties of cross-linked structure are not only related to the properties of epoxy resin matrix, curing agent and filler itself, but also closely related to the formation conditions of cross-linked structure. The curing system of epoxy resin can be summarized as the proportion of matrix and curing agent, the proportion of accelerator, and the combination of curing temperature and time.

The optimal ratio of epoxy resin and curing agent has been studied fully. Alhabill et al. studied the influence of the ratio of bisphenol-A epoxy resin DER332 and amine curing agent D-230 on the insulation performance. The results showed that although the DC conductivity, dielectric property and AC/DC breakdown mechanism were different, most of the solidified products prepared according to the theoretical reaction ratio obtained the best performance. The maximum glass transition temperature ($T_{g}$) appears at the optimum ratio, which is due to the change of crosslinking density. The DC conductivity depends on the chemical composition of the system and is less affected by the network structure. When the curing agent is excessive, the conductivity of the cured sample increases significantly, which may be related to the content of residual amino groups; The maximum DC breakdown strength is obtained at the equimolar ratio, which can also be explained by the difference in network structure [67]. Nguyen et al. studied the influence of the ratio of bisphenol A epoxy resin and anhydride curing agent HY906 on the thermal stability and DC breakdown strength of pure epoxy and nano SiO_2_ filler system. No matter whether filler is added or not, the epoxy system shows the maximum $T_{g}$ at the equimolar ratio, and the results of the breakdown strength are slightly different. The breakdown field strength of pure epoxy system is the maximum at the equimolar ratio, reaching nearly 173 kV/mm. However, after adding $SiO_{2}$ filler, the maximum breakdown field strength is at the position where the epoxy matrix is slightly excessive, which indicates that the addition of filler requires compensation calculation for the optimal reaction ratio [68]. Vryonis et al. recalculated the optimal ratio of bisphenol A epoxy resin DGEBA and anhydride curing agent MTHPA according to the principle of equal proportion reaction in the presence of epoxy diluent octaglycidyl ester (OG) and studied the thermal stability and dielectric properties. The best ratio after compensation shows the best $T_{g}$. Although the dielectric constant of the sample with excessive curing agent is the lowest, the sample with the best ratio after compensation shows lower α and β relaxation peak strength [69]. Li et al. used quantum chemical calculation to clarify the mechanism of space charge characteristics in the amine-type hardener cured epoxy and anhydride-type hardener cured epoxy. It is indicated that ionization of remnant unreacted amine-type hardener and electrode injection may be the main sources of space charge [10]. In summary, according to the reaction principle, the theoretical mass ratio calculated based on the molar ratio reaction of epoxy group and anhydride group is the optimal ratio of epoxy resin and curing agent. Excess epoxy or curing agent will affect the formation of a dense cross-linking network, which will have adverse effects on the properties of curing products.

Amirova et al. studied the thermal performance of phosphine salts as promoters for anhydride cured epoxy resin samples and found that 3–4 wt% is the best proportion of phosphine salts promoters, which is conducive to the formation of three-dimensional cross-linking networks with the least defects [70]. Gou et al. developed a latent accelerator for an epoxy/anhydride system encapsulating triphenylphosphine (TPP). The accelerator improves the glass transition temperature of epoxy thermosetting resin-based on the organic-inorganic hybrid mechanism and has toughening effect [71]. However, most of these studies focus on the thermal and mechanical properties of epoxy resin, and less on the insulation properties. Li et al. found the content of accelerator has little effects on the bulk conductivity and breakdown field strength of DEBGA/MHHPA system but has obvious effect on the dielectric properties. The epoxy resin has the best insulation performance when the accelerator content is 0.5 wt%. The increase of energy band gap of cured epoxy resin is an important reason for the increase of electrical conductivity under the action of a small amount of accelerator and an excessive amount of accelerator [41]. 

The difficulty in the study of curing temperature and time is that there are many combinations, and there is a lack of corresponding theoretical basis and selection criteria. At the same time, suppliers of chemical raw materials generally provide experienced curing schemes, so there is not much research in this area. Saeedi et al. studied the DC conductivity, AC breakdown and dielectric properties of bisphenol A epoxy DGEBA and amine curing agent D-230 at 80 °C for different curing times (1–9 h) and at 80 °C for 2 h, and at 125 °C for different post curing times (1–3 h). The results showed that the samples cured at 80 °C for 6 h had the highest $T_{g}$, the conductivity increased first and then decreased with the increase of curing time, and the trend of AC breakdown field strength was opposite, while the extreme values appeared at the position of curing time for 3 h, and the dielectric constant and dielectric loss reached the maximum at 6 h of curing time, and then gradually decreased. In comparison, the change of post curing time has no obvious effect on the properties of samples [72]. The influence of curing time on the formation of epoxy resin structure is complex, and the longer the curing time is, the better the performance is. Uzay et al. studied the effect of post curing heat treatment on the tensile and impact toughness of fiber-reinforced polymer (FRP) composites, and the results show that increasing the post curing temperature from 25 to 100 °C resulted in 12%, 7.1%, and 8.9% increase in tensile strength. The increase for the impact toughness of carbon, glass and hybrid FRP composites was 10.5%, 9.7%, and 10.6%, respectively [73]. Guerrero et al. studied the relationship between the dynamic mechanical behavior of TGDDM/THPA epoxy system and curing time and stoichiometry. The curing system was 120 °C 2 h + 170 °C 2 h + 200 °C 2 h. The results show that post curing improves the $T_{g}$ and mechanical properties of epoxy resin, which is attributed to the etherification of unreacted epoxy groups by post curing, which makes the cross-linking structure more uniform [74]. In order to compare the effect of different post curing treatment time on the reaction process and insulation performance of the DEBGA/MHHPA system, 80% and 90% of the pre-curing process (80 min or 123 min at 100 °C) were selected. On this basis, 140 °C post-curing was carried out for 1 h, 2 h, 4 h and 8 h, respectively, to investigate the impact of post curing on insulation performance. It can be found in Table 4 that the insulation properties of epoxy resins post-cured on the 90% curing degree basis were generally better than those of post-cured samples on the 80% curing degree basis and the influence of post curing time has saturation effect. The insulation properties were evaluated comprehensively, and the optimal curing regime was finally selected as 0.5% DMP-30 + 123 min curing at 100 °C + 4 h post-curing at 140 °C [36].

## 6. Conclusions

(1) The results of the model fitting curing kinetics showed that the DEBGA/MHHPA system meets the *n*-order reaction model and Kamal model before and after the curing degree of 60%, which indicates that the reaction rate affected by concentration and catalytic effect is increasing. The staged model fitting is a solution to accurately describe and understand the curing kinetics of the DEBGA/MHHPA system.

(2) The epoxy resin forms a three-dimensional network structure through the ester group and ether bond as the crosslinking point. With the progress of crosslinking reaction, the occupied volume decreases, the free volume and the proportion of free volume first decrease and then increases, the total energy of the system decreases, and the molecular bonding is tighter. The electronic affinity of the overall molecular structure and the energy band gap can help to explain the effects of molecular structure on the insulation performance.

(3) The curing regime directly affects the operational reliability of insulating components of electrical and electronic equipment. Continuous optimization of the curing regime has scientific and engineering significance for the development of high-performance epoxy-based insulating materials.

## Figures and Tables

**Table 1:** Characteristic temperature of DEBGA/MHHPA system with different accelerator contents [41].

DMP-30 (wt%)	*T*_i_/(°C)	*T*_p_/(°C)	*T*_t_/(°C)
0.1	102.3	144.2	167.7
0.2	96.5	139.85	164.1
0.5	95.8	138.1	159.8
1	92.1	134.6	156.55

The initiation temperature (*T*_i_), the peak temperature (*T*_p_) and the termination temperature (*T*_t_) can be obtained from the DSC (Differential Scanning Calorimetry) curves.

**Table 2:** Reaction activation energies of DEBGA/MHHPA system with various DMP-30 contents [41].

DMP-30 (wt%)	Kissinger Method	Friedman Method	F-W-O Method
	$\ln\frac{\beta }{T_{p}^{2}}=\ln\frac{AR}{E_{a}}-\frac{E_{a}}{RT_{p}}$	$\ln(\frac{d\alpha }{dt})=-\frac{E_{a}}{RT}+\ln(Af(\alpha ))$	$\ln(\beta )=-\frac{1.052E_{a}}{RT}+\ln(A)-\ln g(\alpha )-5.331$
0.1	93.67	85.403	95.894
0.2	75.947	77.392	79.032
0.5	73.781	75.412	76.921
1	70.99	65.861	74.102

*E*_a_ represents activation energy, *β* represents heating rate, *A* represents frequency factor and *R* represents gas constant.

**Table 3:** Model-fitting curing kinetics of DEBGA/MHHPA system with 0.2 wt% DMP-30 [41].

Parameter	Kamal Model	*n-*Level Reaction Model
	$\frac{d\alpha }{dt}=(k_{1}+k_{2}\alpha^{m}){\left(1-\alpha \right)}^{n}$	$\alpha (t)=1-{\left[1-(1-n)Ae^{-\frac{E_{a}}{RT}}t\right]}^{\frac{1}{1-n}}$
*k*_1_	0.0275	/
*k*_1_	0.3008	/
*m*	0.4912	/
*n*	1.5060	/
*E_a_*	/	75.9469
*n*	/	0.9135
*A*	/	7.2839 × 10^8^

**Table 4:** Effects of post-curing process on the breakdown characteristics of DEBGA/MHHPA system under 100 °C [36].

Post-Curing Process	Parameter *α* (kV/mm)	Parameter *β*
80% + 1 h	50.72	13.55
80% + 2 h	51.83	16.83
80% + 4 h	54.92	10.22
80% + 8 h	53.11	8.956
90% + 1 h	52.30	10.84
90% + 2 h	54.07	14.66
90% + 4 h	56.85	11.18
90% + 8 h	55.48	15.58

## Data Availability

Not applicable.

## Sample Availability

Samples of the compounds are not available from the authors.
